# Adverse effects of COVID-19 pandemic on a multicenter randomized controlled trial

**DOI:** 10.1038/s41372-022-01592-2

**Published:** 2022-12-29

**Authors:** Elie G. Abu Jawdeh, Carl E. Hunt, Eric Eichenwald, Michael J. Corwin, Betty McEntire, Timothy Heeren, Lisa M. Crowell, Christine Ikponmwonba, Ariana Saroufim, Stephen Kerr, the ICAF Study Group, Robert Darnall, Christian Poets, Mary Revenis, Melissa Tyree, Ann Pokelsek, Ann Stark, Ivan Frantz, Neha Thakkar, Rachel Hansen, Toni Mancini, Megan Dhawan, Tyler Hartman, Mary McNally, Karlyn Martini, Prem Fort, Sarah Flanagan, Tamara Babushkin, Haley Sivilich, Venkataraman Balaraman, Micah Tong, Mitchell Goldstein, Tina Ramirez, Nikia Gray-Hutto, Ashra Tugung, Kimberly Quire, Sara Butler, Carrie Hobbs, Lawrence Rhein, Anjana Shenoy, Lindsey Simoncini, Jaimin Patel, Heather Williams, Chelsea Giachelli, Nicole Dobson, Elizabeth Schulz, Judith Fitzpatrick

**Affiliations:** 1 University of Kentucky, Lexington, KY USA; 2 Uniformed Services University, Bethesda, MD USA; 3 Children’s Hospital of Philadelphia and University of Pennsylvania, Philadelphia, PA USA; 4 Boston University, Boston, MA USA; 5 American SIDS Institute, Naples, FL USA; 6 Dartmouth University, Hanover, NH USA; 7 University of Tuebingen, Tuebingen, Germany; 8 Children’s National Hospital, Washington, DC USA; 9 George Washington University, Washington, DC USA; 10 AdventHealth for Children, Orlando, FL USA; 11 Beth Israel Deaconess Medical Center, Boston, MA USA; 12 Dartmouth Hitchcock Memorial Hospital, Lebanon, NH USA; 13 Johns Hopkins All Children’s Hospital, St. Petersburg, FL USA; 14 Kapiolani Medical Center for Women and Children, Honolulu, HI USA; 15 University of Hawaii, Honolulu, HI USA; 16 Loma Linda University Children’s Hospital, Loma Linda, CA USA; 17 University of Massachusetts, Worchester, MA USA; 18 University of Mississippi Medical Center, Jackson, MS USA; 19 Walter Reed National Military Medical Center, Bethesda, MD USA

**Keywords:** Medical research, Scientific community

## Abstract

**Objective:**

Describe the effects of the COVID-19 pandemic on subject enrollment in a multicenter randomized controlled trial.

**Study design:**

We assessed the number of eligible infants approached and consented for enrollment over five separate epochs including baseline, peak pandemic, and gradual but incomplete recovery.

**Result:**

The pandemic had a major effect on ability to approach parents for consent. Parents approached dropped from 95.4% baseline to 13.1% in the peak pandemic epoch and has not recovered to baseline even in the just-completed post-pandemic epoch (84.9%). Despite the decrease in subjects approached, there was no significant change in the overall consent rate for the study

**Conclusion:**

The pandemic has significantly limited ability to approach parents of eligible infants for consent, with only partial recovery. Opportunities for interactions of investigators and study coordinators with parents continue to present challenges limiting full recovery.

## Introduction

The COVID-19 pandemic has significantly disrupted family and community stability and well-being [1]. The successful conduct of clinical research depends on multiple factors including suitable numbers of subjects and demographics, risk to benefit ratio, study burden, and sufficient access to approach eligible subjects. The pandemic likely increased stress on health care providers and families, and decreased the research activity of investigators and study coordinators [2, 3]. Importantly, the pandemic also exacerbated the parental stresses typically associated with NICU admission, including visitation restrictions [4].

There is very limited published information regarding the adverse effects of COVID-19 on clinical research. A report of the effect of a one-parent/visitor policy on the parental consent rate during the acute pandemic months (May-July, 2020) compared to pre-pandemic baseline observed that this restriction on parental visitation reduced the consent rate [5]. However, there are no data assessing the impact of the COVID-19 pandemic on randomized controlled trials and, of particular note, no data on recovery rates or persisting adverse effects after mid-2020. To address this lack of data, we now report the effects of COVID-19 on the Intermittent Hypoxia and Caffeine in Infants Born Preterm (ICAF) study, a multicenter trial that involves both an inpatient and post-discharge protocol.

## Methods

### ICAF protocol

The ICAF Study is an NIH-funded multicenter randomized controlled trial in infants born preterm (NCT03321734), in which convalescent preterm infants treated with caffeine are randomized to continued caffeine or placebo at the time of clinical caffeine discontinuation. The primary aims of the study are to compare, from randomization through 42^+6^ weeks postmenstrual age (PMA), 1) the effect of caffeine on extent of intermittent hypoxia (IH), 2) inflammation-related cytokines and chemokines from baseline to 38 weeks PMA, and 3) quantitative MRI structural, microstructural and metabolic biomarkers of injury from baseline to 43–46 weeks PMA. In addition to birth at ≤ 30^+6^ weeks gestation, the inclusion criteria include current routine treatment with caffeine and expected last routine dose at ≤ 36^+5^ weeks, current PMA 32^+0^ weeks to 36^+6^ weeks, no ventilatory support for at least 12 h other than room air nasal airflow therapy or nasal CPAP, able to tolerate enteral medications, and feasible to administer first dose of study drug no later than 36^+6^ weeks PMA. The exclusion criteria include severe intraventricular hemorrhage (Grades 3-4) or cystic periventricular leukomalacia, current or prior treatment for seizures or cardiac arrhythmia, major malformation or congenital or chromosomal abnormality, or social or other issues precluding successful parental compliance.

Beginning at enrollment at 32^+0^ weeks to 36^+6^ weeks, a study pulse oximeter (Masimo RAD-97, modified to provide up to 28 days of data storage) is used continuously in the NICU and during sleep and quiet awake time at home. The study drug is caffeine base at an enteral dose of 5 mg/kg/dose or equal volume placebo, administered daily until 36 weeks PMA and then twice daily thereafter until 42^+6^ weeks. A baseline quantitative MRI/MRS is obtained as soon after enrollment as possible, but no later than 35 weeks PMA. To measure pro- and anti-inflammatory biomarkers, a baseline blood sample (0.6–0.8 ml) is obtained at the time of randomization and starting study drug at ≤ 36^+6^ weeks PMA, and the second sample obtained at 38 weeks PMA or within the last 2 days prior to discharge, whichever comes first. Whenever feasible, the blood samples are obtained as part of a routine clinical blood sample. An inpatient salivary sample for later measurement of caffeine concentration is collected about 7 days after beginning twice daily dosing of study drug [6, 7].

The study protocol continues at home for several weeks, the duration depending on PMA at NICU discharge. Twice daily administration of the enteral study drug is continued at home until 42^+6^ weeks PMA. The pulse oximeter is used daily during all sleep time until discontinued at 43^+6^, one week after stopping study drug. As previously instructed, the parents obtain a salivary sample at about 40^+0^ weeks PMA but not later than the last day of study drug. The Brief Infant Sleep Questionnaire is completed by phone interview during the last week of receiving study drug [8]. The family returns to the medical center no later than 46^+6^ weeks PMA for the follow-up MRI.

The target sample size is 220 subjects, including at least 200 with complete data for analysis. Based on prior experience, we estimated that 40% of all infants born at ≤ 30^+6^ weeks gestation and added to the screening log would be eligible to approach for consent, and 25% of these would be successfully consented. A total of 8 medical centers was included at onset of this study. We added four additional NICUs prior to the pandemic, and three more clinical sites as clinical research resumed after the peak pandemic period. Enrollment is scheduled to continue until June 1, 2023. The Institutional Review Board at each site approved the study.

### Analyses of pandemic effects

Despite the onset of the pandemic in March, 2020, all clinical sites were able to continue entering infants in the screening log. However, only one site was permitted to continue any other clinical research activity during the peak pandemic epochs, including parent interactions and approaching for consent. The data coordinating center (DCC) tracks the number of infants who become eligible, are approached for consent, and who are consented and enrolled. The DCC also captures available information as to why an eligible patient could not be approached (e.g.: staff or family not available) or the apparent reason for parental refusal of consent (e.g.: study procedures too burdensome, or parent does not want to participate in research). For purposes of this analysis, we defined five study epochs to be representative of the sequential periods of pandemic-related effects, beginning December 31, 2018 and ending July 22, 2022. The pre-COVID epoch (12/31/18-3/5/20) represents the baseline screening and enrollment activity from initial enrollment until pandemic onset. The COVID-1 epoch (3/6/20- 7/31/20) includes the lockdown period when clinical sites severely restricted or suspended all clinical research activity except for screening, and the COVID-2 epoch (8/1/20- 3/5/21) reflects the beginning recovery in clinical research activity. The Post-COVID-1 epoch (3/6/21- 11/11/21) represents continuing recovery from the peak adverse pandemic effects, and Post-COVID-2 (11/12/21- 7/22/22) represents our final observed epoch of continuing recovery in study conduct.

During the peak pandemic epoch and beginning recovery, several site-specific actions were approved by multiple site IRBs to improve consent rates. These included approval for telemedicine-based or REDCap-based consents, and approval for early consent for subjects potentially eligible for enrollment but not yet satisfying all criteria required for enrollment and for randomization.

### Statistical analyses

Baseline characteristics of eligible families and infants are described using percentages for categorical characteristics and means (standard deviations) for continuous characteristics. Associations between demographic characteristics of those approached vs. not approached, and between those who were approached and enrolled vs. refused enrollment, are tested using chi-square tests for categorical characteristics and t-tests for continuous characteristics. The enrollment process over the study period is described through the cumulative number eligible and cumulative number approached, and approach rates are compared between study epochs through chi-square tests. Multiple logistic regression is used to compare both approach and consent rates across study epochs, controlling for characteristics found to be associated with being approached in earlier analyses, which included maternal race/ethnicity for analysis of approach rate, and maternal race/ethnicity and gestational age at birth for consent rates.

## Results

Over the three and a half years of screening, 2760 infants have been screened and had their eligibility determined (Table 1). A total of 774 infants (28% of those screened) was eligible for the study. Among the eligible infants, the mean gestational age (GA) and birth weight are 28^+5^ weeks and 1180.6 g, respectively. The families of eligible subjects approached for consent differ significantly on race/ethnicity from those families not approached (*p* = 0.047). Compared to the 32.1% not-approach rate for consent in the non-Hispanic (NH) White reference group, for example, 44.9% of Hispanic families were not approached. Non-approach rates for NH Blacks (29.8%) did not differ from the NH White group. Of families who were approached, consent rates varied significantly among NH Whites (28.5%), NH Blacks (20.3%), Hispanics (8.2%), and NH other (12.8%), *p* = 0.001.

**Table 1:** Maternal and infant characteristics for all subjects eligible to be approached for consent in the ICAF Study, subjects enrolled, parents of eligible infants approached but refusing consent, and all those eligible overall, but not approached for consent.

Characteristics	All Eligible *N* = 774	Enrolled *N* = 110	Approached but Refused *N* = 413	Not Approached *N* = 251
MATERNAL				
Mean Age in years (SD)	30.5 (6.2)	30.6 (6.6)	30.4 (5.9)	30.6 (6.4)
Race/ethnicity*, *n* (%)				
Hispanic	89 (11.5)	4 (3.6)	45 (10.9)	40 (16.1)
NH-Black	245 (31.8)	35 (31.8)	137 (33.3)	73 (29.3)
NH-White	305 (39.6)	59 (53.6)	148 (35.9)	98 (39.4)
All Other	132 (17.1)	12 (10.9)	82 (19.9)	38 (15.3)
INFANT, *n* (%)				
Male	382 (49.4)	45 (40.9)	210 (50.8)	127 (50.6)
Female	392 (50.6)	65 (59.1)	203 (49.2)	124 (49.4)
Birthweight (grams)				
Mean (SD)	1180.6 (313.6)	1144.8 (328.9)	1186.9 (295.1)	1185.7 (335.6)
Median (Min-Max)	1170 (430–2120)	1128 (430–1895)	1179 (470–2000)	1160 (470–2120)
Gestational age (GA) at birth				
Mean GA in weeks, SD (days)	28 + 5 (11.7)	28 + 2 (12.0)	28 + 6 (10.9)	28 + 5 (12.7)

*ICAF* Intermittent hypoxia and caffeine in infants born preterm study. All Other: includes Non-Hispanic (NH) American Indian/Alaska Native, NH Asian, NH Native Hawaiian/Pacific Islander, NH Other. *N* (% of column total) unless otherwise specified. *There were differences in race/ethnicity among approached and enrolled infants, all *p* < 0.05.

### Effect of pandemic on approaching parents for consent

**Fig. 1:**
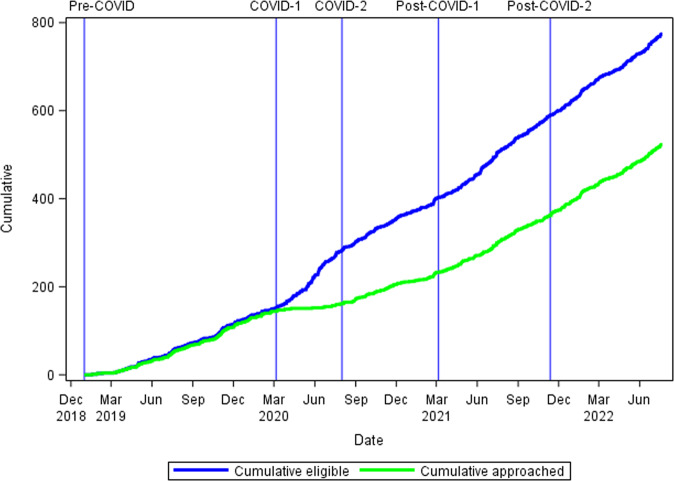
Cumulative numbers of infants eligible for enrollment and families of eligible infants approached for consent in the Pre-COVID-19 baseline epoch; COVID-1 epoch; COVID-2 epoch; Post-COVID -1 epoch; and Post-COVID-2. The blue line represents the cumulative increase in the number of infants eligible for enrollment, and the green line represents the cumulative number of parents of eligible infants approached for consent. In the Pre-COVID epoch, 95.4% of parents of eligible infants were approached for consent. From the nadir in COVID-1, the cumulative number of parents of eligible infants approached progressively improved in the subsequent epochs, but only to 84.9% in Post-COVID-2 (*p* < 0.001 each subsequent epoch compared to Pre-COVID).

The cumulative number of infants eligible and approached for the study over time is provided in Fig. 1. As noted in Methods, screening activities continued even when enrollment was prohibited. The number of eligible patients increased over time and as more clinical sites were added. During the Pre-COVID epoch, research staff were able to approach the families of 144 of 151 (95.4%) eligible infants. During the initial pandemic lockdown (COVID-1 epoch), only 17 of 130 (13.1%) of eligible families could be approached for consent (AOR 0.007; 95% CI 0.003, 0.017 compared to the Pre-COVID epoch). This accounts for the divergence of the slopes of eligible and approached subjects seen in Fig. 1. The percent of eligible families approached for consent has gradually improved since the COVID-1 epoch: 72 of 122 (59.0%, AOR 0.1; 95% CI 0.0, 0.2), 132 of 185 (71.4%, AOR 0.11; 95% CI 0.05, 0.26) and 158 of 186 (84.9% AOR 0.3; 95% CI 0.1, 0.7) over the COVID-2, Post COVID-1 and Post COVID-2 epochs, respectively, but remains significantly less than in the Pre-COVID epoch even in the Post-COVD-2 epoch ending in July, 2022. Differences in approach rates across study epochs, compared to pre-COVID, remain significant after controlling for race/ethnicity through multiple logistic regression.

**Fig. 2:**
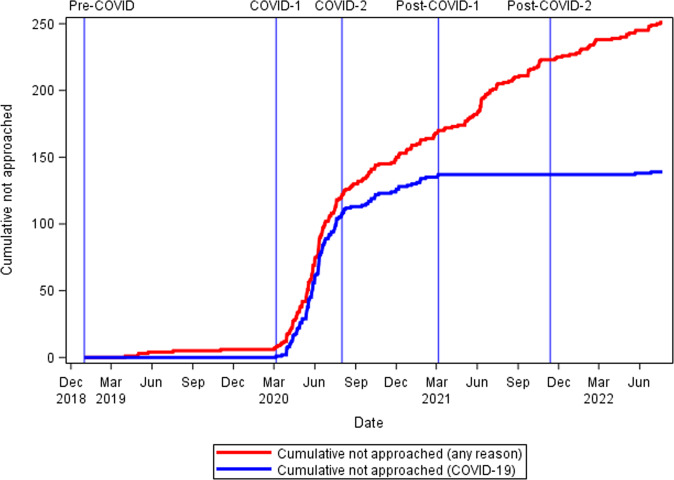
Cumulative number of parents of eligible infants not approached for consent in the Pre-COVID-19 epoch (baseline); COVID-1 epoch; COVID-2 epoch; Post-COVID -1 epoch; and Post-COVID-2. The red line represents the cumulative number of parents not approached for any reason, and the blue line represents parents not approached due directly to COVID-19-related restrictions on clinical research. These site-specific restrictions gradually abated in COVID-2 and were not a factor in Post-COVID epochs. The cumulative number of all families not approached for any reason has continued to increase even in the two Post-COVID epochs, as the percent of families of eligible infants approached for consent has still not fully recovered.

**Fig. 3:**
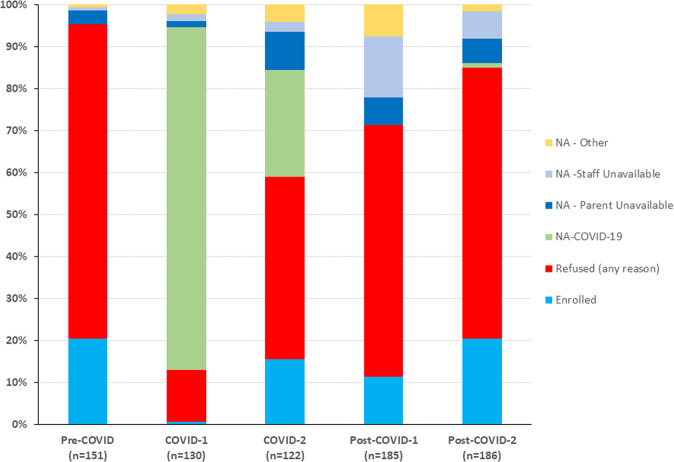
Stacked bar graphs illustrating the relative changes in percentages of eligible infants enrolled, eligible infants approached but consent refused (any reason), and families not approached (NA) for consent, related to COVID-19 restrictions on clinical research, parent or staff unavailability or other reason. Data are summarized across five study epochs: Pre-COVID-19 epoch (baseline); COVID-1 epoch; COVID-2 epoch; Post-COVID -1 epoch; Post-COVID-2 epoch. The sum of the enrolled plus refused equals the total percent of all families of eligible infants approached for consent. The height of each segment compared to the same segment in other columns reflects the relative change in that variable across the epochs. Of particular note, the sum of the four NA subgroups (15.1%) remains significantly increased compared to only 4.6% in the baseline Pre-COVID epoch.

The COVID-1 epoch saw a dramatic increase in number of eligible families not approached due to clinical site-imposed restrictions on research (Fig. 2). In the COVID-2 epoch, the increase in non-approaches for COVID-related reasons progressively diminished as increasing numbers of sites were being allowed to resume clinical research. Clinical site-related limitations on availability of research staff including study coordinators in the Post-COVID-1 and Post-COVID-2 epochs are continuing to limit the cumulative number of parents of eligible infants being approached for consent (also see Fig. 3).

Figure 3 illustrates all of the reasons why families of eligible infants were not being approached for consent and delineates the several reasons for non-approach in addition to site-specific restrictions on clinical research. Whereas staff or parent unavailability accounted for non-approaches in only 4.0% of eligible families in the Pre-COVID epoch and 3.1% in COVID-1, this increased dramatically to 11.5% in COVID-2. Parent and staff unavailability account for 21.1% and 12.4% in Post-COVID-1 and Post-COVID-2, respectively and represent the primary factor in explaining the inability to achieve the same percentage of eligible subjects being approached for consent as were being approached in the baseline Pre-COVID epoch (95.4%).

### Consent rates in families approached for consent

The consent rate in parents of eligible infants approached for consent has not changed significantly from the Pre-COVID epoch through Post-COVID-2. The dramatic decrease in the percent consented during COVID-1 epoch was not statistically significant. During the Pre-COVID epoch 31 of 144 (21.5%) families approached provided consent. In comparison to the Pre-COVID period, the consent rates for the subsequent four study epochs are: one of 17 (5.9%, AOR 0.2; 95% CI 0.0, 1.8), 19 of 72 (26.4%, AOR 1.4; 95% CI 0.7, 2.8), 21 of 132 (15.9%, 0.8; 95% CI 0.4, 1.4) and 38 of 158 (24.1%, AOR 1.3; 95% CI 0.7. 2.2), respectively.

## Discussion

Compared to the baseline Pre-COVID epoch, the conduct of the ICAF study has been significantly and adversely affected by the pandemic. To illustrate the progressive pandemic effects, we defined five epochs, the first prior to pandemic onset, and four subsequent epochs, with the Post-COVID-2 epoch ending 07/22/22. Sites were able to continue screening new admissions and adding potentially eligible subjects to the database during the peak pandemic, but the primary adverse effect of the pandemic has been limitations on approaching the parents of eligible subjects for consent. Although able to approach 95.4% in the Pre-COVID epoch, approach rates fell precipitously during the COVID-1 epoch to 13.1% due to site-specific restrictions on clinical research. From this nadir, the cumulative number of approached families (Fig. 1) has progressively increased, but only to 84.9%. After allowed to resume clinical research, the primary obstacles to approaching families to ask for consent are limitations on research staff or parent availability. Unexpectedly, these limitations remain, albeit to a significantly lesser extent than during the peak pandemic. Of note, however, the pandemic has not adversely affected the percent of approached families who consented to enroll in the study compared to the Pre-COVID epoch.

Published data on COVID-19-related effects on clinical research are very limited. Van Driest et al. reported the changes in consent rates before and during a COVID-19-imposed one-visitor policy in a single children’s hospital [5]. In this observational study in children scheduled for congenital heart surgery, during the four peak pandemic months of March-July, 2020, enrollment rates decreased significantly to 55% from 71% during the baseline months (*p* = 0.009). Unlike our results, however, the percent of eligible subjects approached for consent remained unaffected (*p* = 0.215). Of note, there was an age-related differential effect on consent rates, with the consent rate for neonates and infants significantly less during the peak pandemic months compared to baseline (*p* = 0.002), with no significant change in children and adults. They speculated that pandemic-related psychological stress as well as the one-visitor policy were significant factors. Unlike our study design, they did not include any data following the peak pandemic months.

The only other report of pandemic effects focused only on health care workers in 11 hospitals and clinics during the first wave of COVID-19 [2]. They observed that, for health care workers reassigned during the wave of COVID-19, their well-being and intent to stay were more favorable if provided the choice to accept or decline the reassignment. Although not directly applicable to our observations, the reassignments of study coordinators in our report may have contributed to perceived workplace-related difficulties in maintaining optimal work environments among remaining study coordinators, and hence possibly affecting study coordinator performance in our later observed epochs.

The marked decrease in our approach rate in the COVID-1 epoch is due to total restrictions on clinical research in almost all of our clinical sites. As these restrictions were gradually lifted, the reasons for our observed failure of approach rates to return to baseline can be attributed primarily to persisting limitations on research personnel in the workplace. Site investigators were initially allowed onsite only when on service, study coordinators were required to work remotely and sometimes with required furloughs. In addition, parent visitation was restricted and often limited to one parent at a time. Finally, many of our experienced study coordinators were reassigned to pandemic-related projects or resigned and, even when sites were permitted to hire new study coordinators, the recruitment process progressed slowly due to slow recovery in normal administrative functions overall. Even though all but two research coordinator positions are filled as of the end of the Post-COVID-2 epoch, the experience levels of newer coordinators overall are not equivalent to the Pre-COVID epoch. Indeed, prior to the onset of the Pre-COVID epoch, senior ICAF staff conducted onsite training orientations at each new site, but no onsite training visits have been conducted since the beginning of the COVID-1 epoch. Since then, all new site orientations and training of research coordinators have been conducted virtually, even for inexperienced coordinators and any coordinators new to the ICAF study. Pandemic-related burdens on the family may be another factor adversely affecting optimal interactions between research staff and parents. The burdens that parents of very preterm neonates in the NICU typically have are likely increased by persisting pandemic-related burdens of parents and in the home environment. Despite these multiple limitations on available study personnel and available families, the percent of parents of eligible infants approached for consent has continued to progressively improve, albeit not yet to pre-pandemic levels.

The primary strength of this report is its generalizability. ICAF is a multisite prospective randomized trial, with racial/ethnic and geographic diversity. The higher non-approach rate observed among Hispanic families is explained, in large part, by the fact that the hospital with the largest number of eligible Hispanic families also had, by far, the longest duration of COVID-related restrictions on clinical research compared to all other sites. Our experiences should therefore be generalizable to other clinical research studies in other sites. The fact that the ICAF multicenter study has both inpatient and outpatient components makes the challenges described in this manuscript relevant to a variety of other randomized trials. On the other hand, the requirements for at-home study drug administration and pulse oximeter recordings may constitute a greater burden on families than some clinical trials. However, our pandemic-related experiences are not being compared to other randomized controlled trials but rather to our Pre-COVID baseline epoch, and burden would likely have its greatest effect on consent rate, which has not been affected.

Our study has several limitations. One limitation is the lack of granular data regarding parental perspectives. There was, for example, no questionnaire administered to parents to collect data on how the COVID-19 pandemic adversely affected their participation in the study as we did not consider it ethically appropriate, for example, to require parents to further justify their non-consent. We are therefore reliant in this analysis on the perceptions of site investigators and study coordinators based on sequential parent communications. Another potential limitation is that our analysis is based on 5 investigator-defined epochs. However, these epochs were chosen to represent the several time periods corresponding to the progressive evolution of the peak and recovery pandemic periods extending until the time of final data analysis, and arbitrary shifting the boundaries of these epochs did not alter the conclusions. Unlike our analysis of approached rates, for which the number of families approached ranged from 122 to 186 during the five epochs, our analysis of consent rates is limited by the relatively small number of families approached for consent during the peak and earlier recovery epochs (only 17 and 72 families approached, respectively), thus we had limited power to assess even moderate changes in consent rates across the five epochs.

In summary, we report our experiences with a multisite clinical trial from the baseline pre-pandemic period through July 22, 2022. In addition to describing the devastating effects of the pandemic onset on study conduct, we delineate the gradual recovery in study activity and describe the ways in which recovery remains incomplete two years following the peak pandemic effects in early-mid 2020. The primary persisting effect is inability to approach all parents of eligible infants for consent. Delineation of the complex factors underlying our reported adverse and persisting consequences of the COVID-19 pandemic on this multicenter study should be generalizable to other studies and other locations and may provide other investigators and funding agencies with helpful information and guidance.

## Data Availability

The datasets generated during and/or analyzed during the current study are available from the corresponding author on reasonable request.
